# Supplementary material for: Intraspecific phenotypic variation in life history traits of Daphnia galeata populations in response to fish kairomones
Source: PeerJ. 2018 Oct 17;6:e5746. doi: 10.7717/peerj.5746 (PMC6195795; doi:10.7717/peerj.5746)
Supplement: Supplemental Information 24 [file peerj-06-5746-s024.docx]

| round | start breeding | end experiment | total number of days | pop | clone | Number of replicates for life history trait analysis (t4-t14) | | Number of replicates for morphometric analysis | |
| --- | --- | --- | --- | --- | --- | --- | --- | --- | --- |
|  |  |  |  |  |  | control | fish | control | fish |
| 1 | 27.07.2015 | 16.09.2015 | 51 | LC | LC3.1 | 15 | 13 | 9 | 10 |
|  |  |  |  |  | LC3.6 | 15 | 15 | 10 | 10 |
|  |  |  |  | J | J2 | 15 | 15 | 10 | 10 |
|  |  |  |  |  | J1 | 15 | 14 | 10 | 10 |
|  |  |  |  |  | J4 | 9 | 13 | 8 | 10 |
|  |  |  |  | G | G3.1 | 14 | 15 | 10 | 10 |
|  |  |  |  |  | G1.11 | 15 | 15 | 10 | 10 |
|  |  |  |  | M | M5 | 15 | 15 | 10 | 10 |
|  |  |  |  |  | M12 | 12 | 13 | 7 | 10 |
|  |  |  |  |  | M6 | 14 | 14 | 10 | 10 |
| 2 | 21.10.2015 | 17.12.2015 | 57 | LC | LC3.5 | 15 | 15 | 10 | 8 |
|  |  |  |  |  | LC3.7 | 10 | 15 | 4 | 10 |
|  |  |  |  |  | LC3.9 | 13 | 15 | 10 | 10 |
|  |  |  |  | J | J3 | 13 | 13 | 10 | 9 |
|  |  |  |  |  | J2.1 | 15 | 15 | 10 | 10 |
|  |  |  |  | G | G1.12 | 15 | 15 | 8 | 10 |
|  |  |  |  |  | G1.6 | 15 | 12 | 10 | 6 |
|  |  |  |  | M | M2 | 15 | 15 | 10 | 10 |
| 3 | 17.05.2016 | 10.07.2016 | 54 | LC | LC3.3 | 14 | 15 | 10 | 10 |
|  |  |  |  | J | J2.4 | 15 | 15 | 10 | 10 |
|  |  |  |  | G | G1.7 | 15 | 15 | 10 | 10 |
|  |  |  |  |  | G2.1 | 15 | 15 | 10 | 10 |
|  |  |  |  | M | M9 | 15 | 13 | 10 | 10 |
|  |  |  |  |  | M10 | 15 | 15 | 10 | 10 |
