# Supplementary figures and images for: Intraspecific phenotypic variation in life history traits of Daphnia galeata populations in response to fish kairomones
Source: PeerJ. 2018 Oct 17;6:e5746. doi: 10.7717/peerj.5746 (PMC6195795; doi:10.7717/peerj.5746)

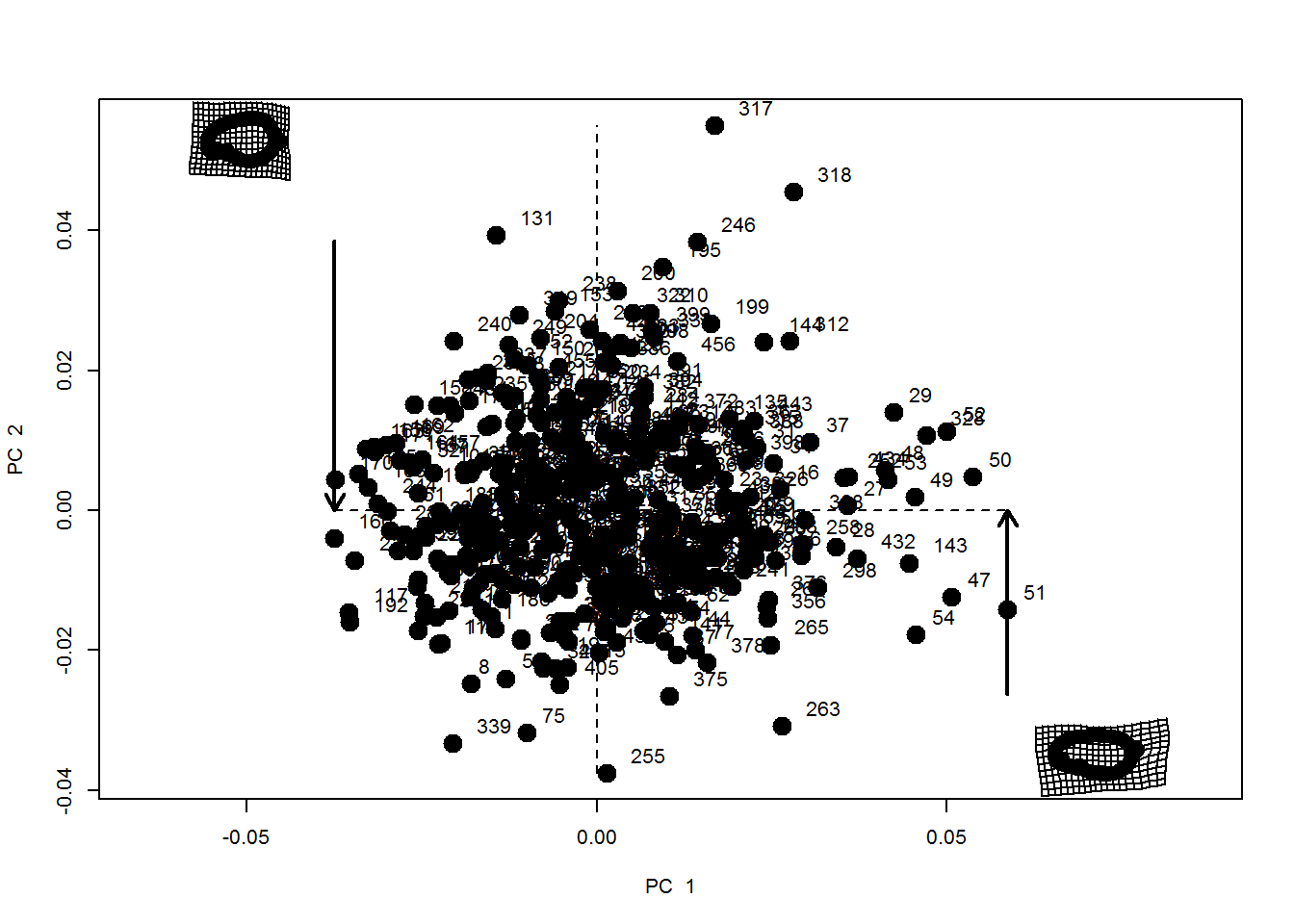

Supplement: Supplemental Information 25 — PC plot of superimposed Procrustes coordinates of all specimen. The thin plate spine grids show shapes associated with the positive end of the horizontal axis and the negative end of the vertical axis. [file peerj-06-5746-s025.png]

A

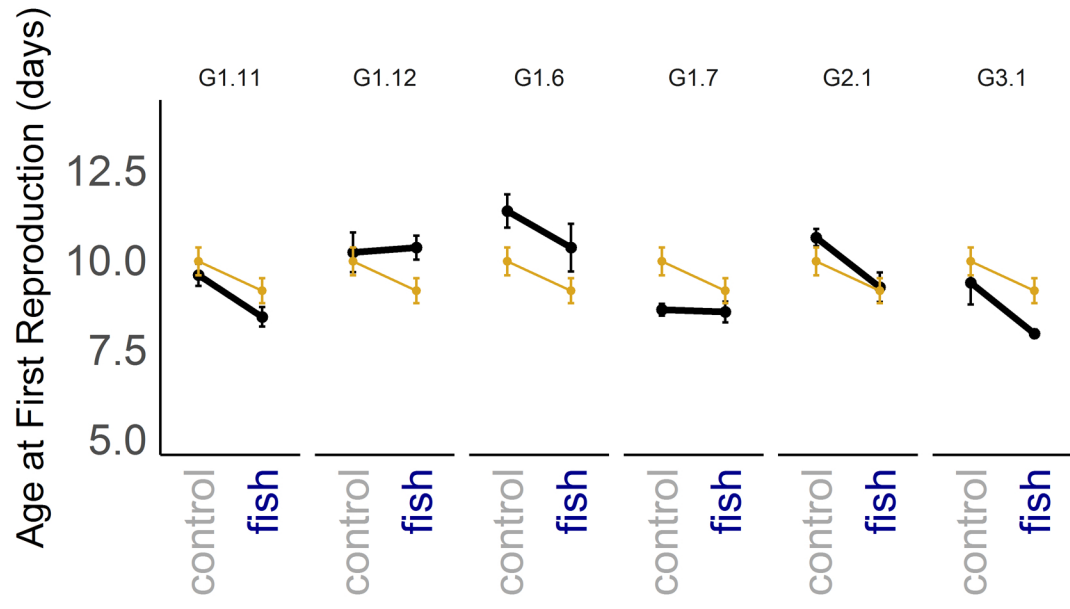

B

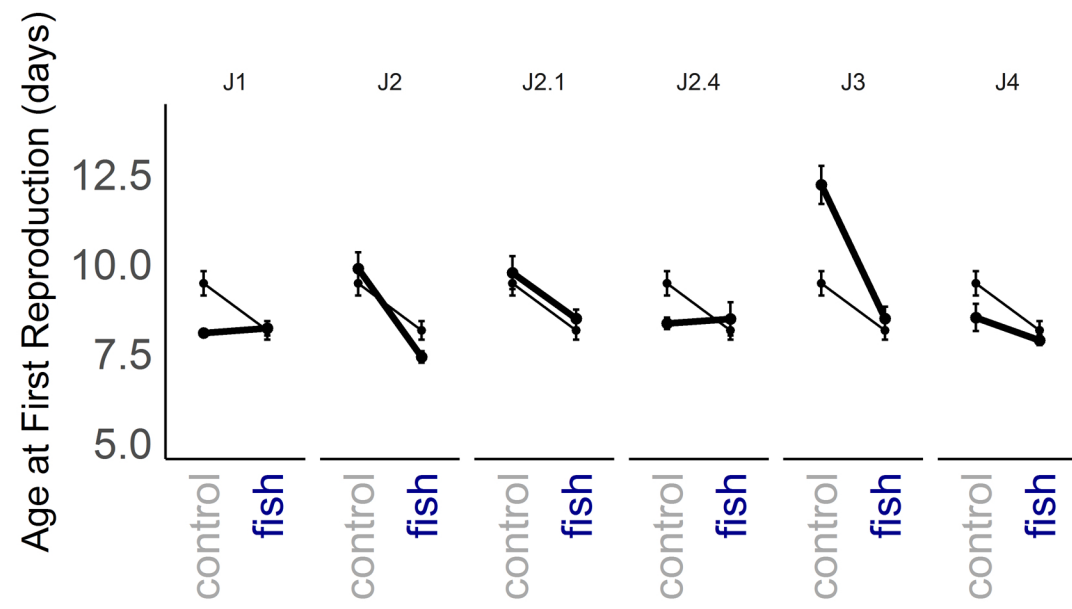

C

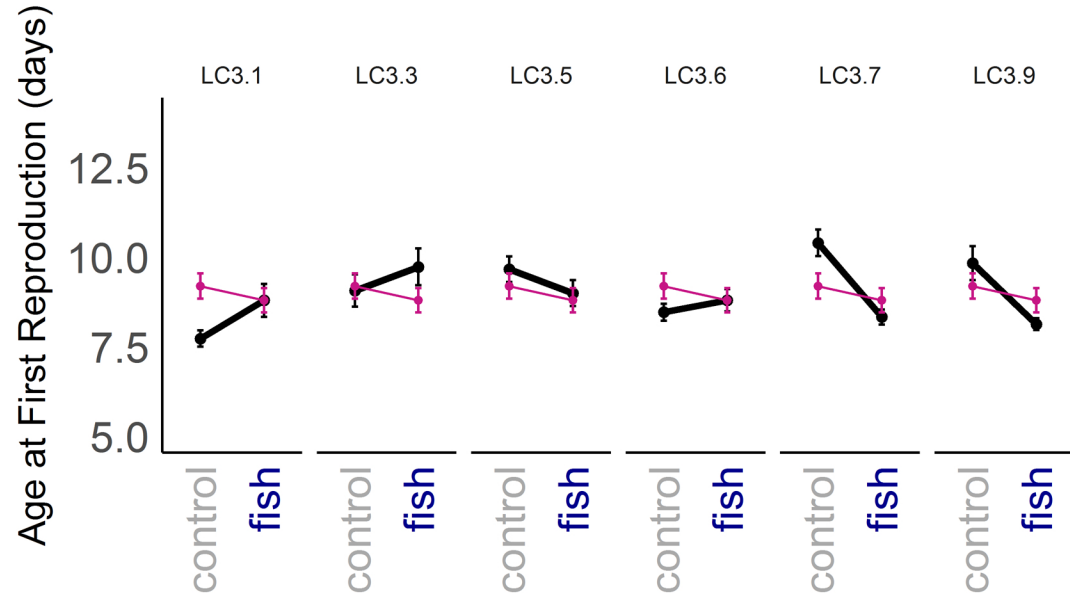

D

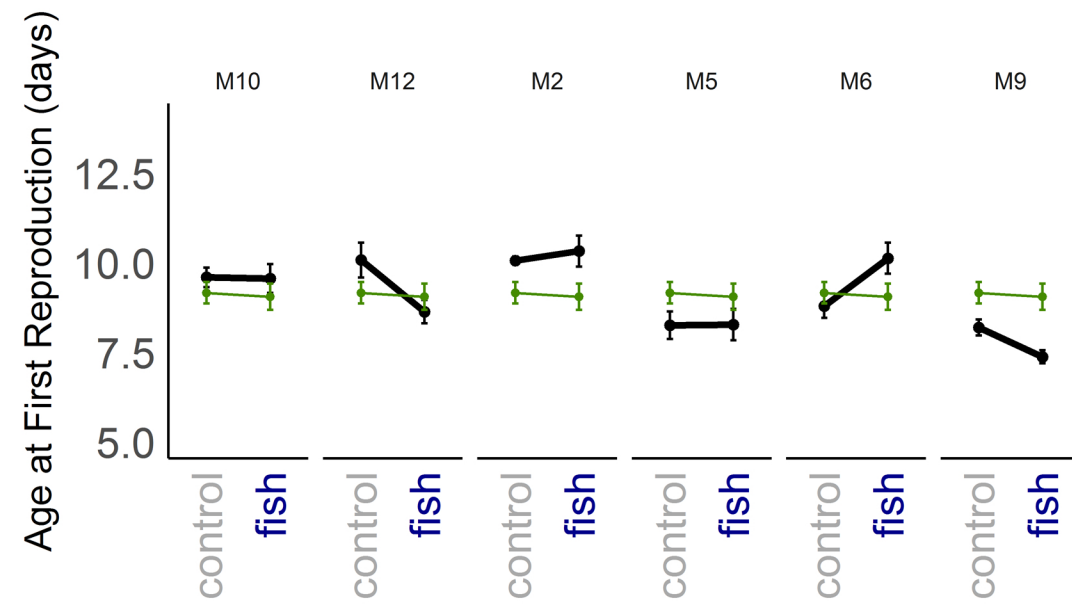

Supplement: Supplemental Information 26 — Genotype mean (+/−SE) within one population are displayed for the trait ‘AFR’ in days. The overall within population mean (+/−SE) is displayed in a population specific color. (A) Population Greifensee= popG= ’yellow.’ (B) Population Jordan Reservoir= popJ= ’black.’ (C) Population Lake Constance= popLC= ’magenta.’ (D) Population Müggelsee= popM= ’green.’. [file peerj-06-5746-s026.pdf]

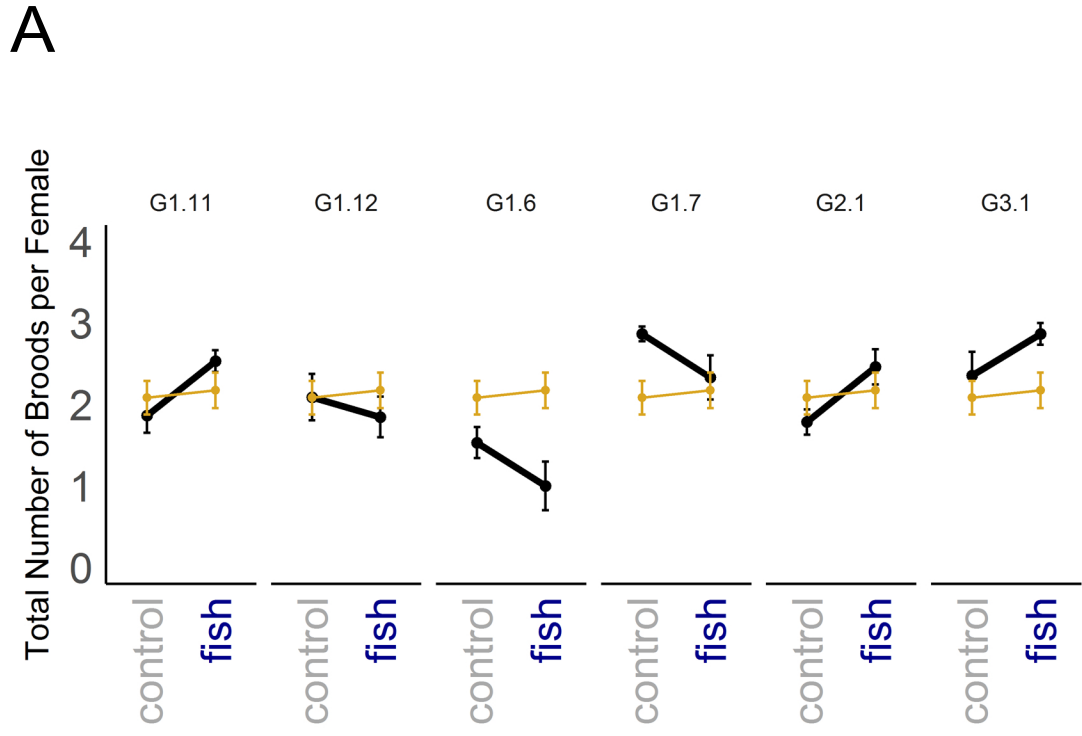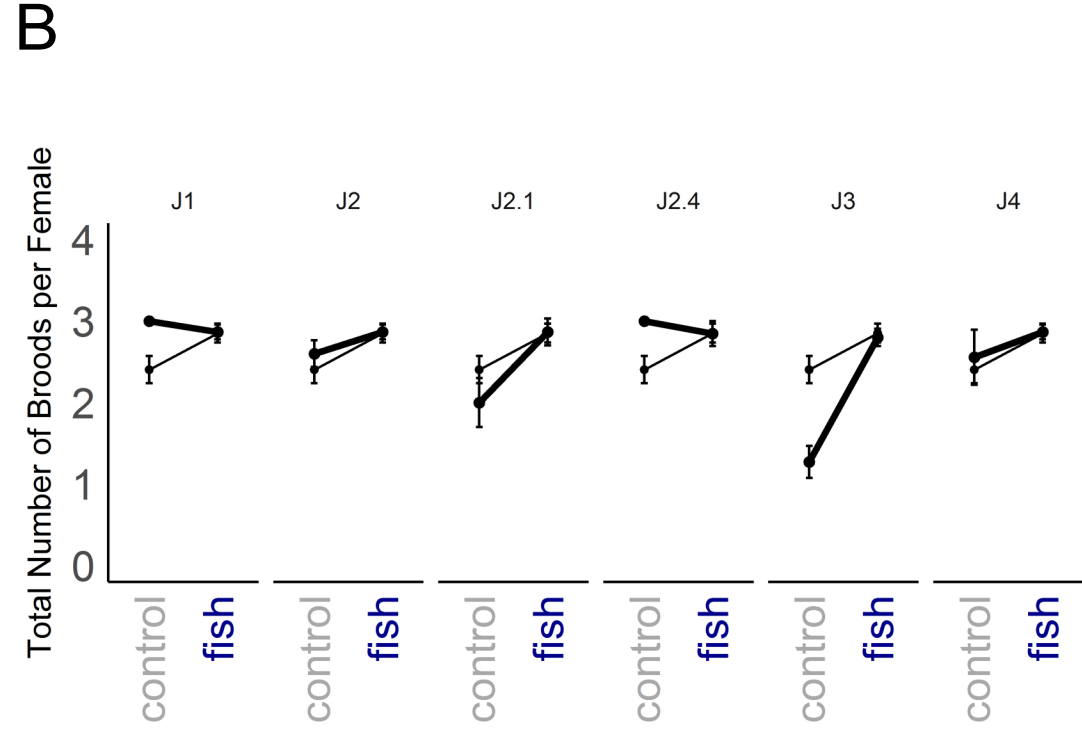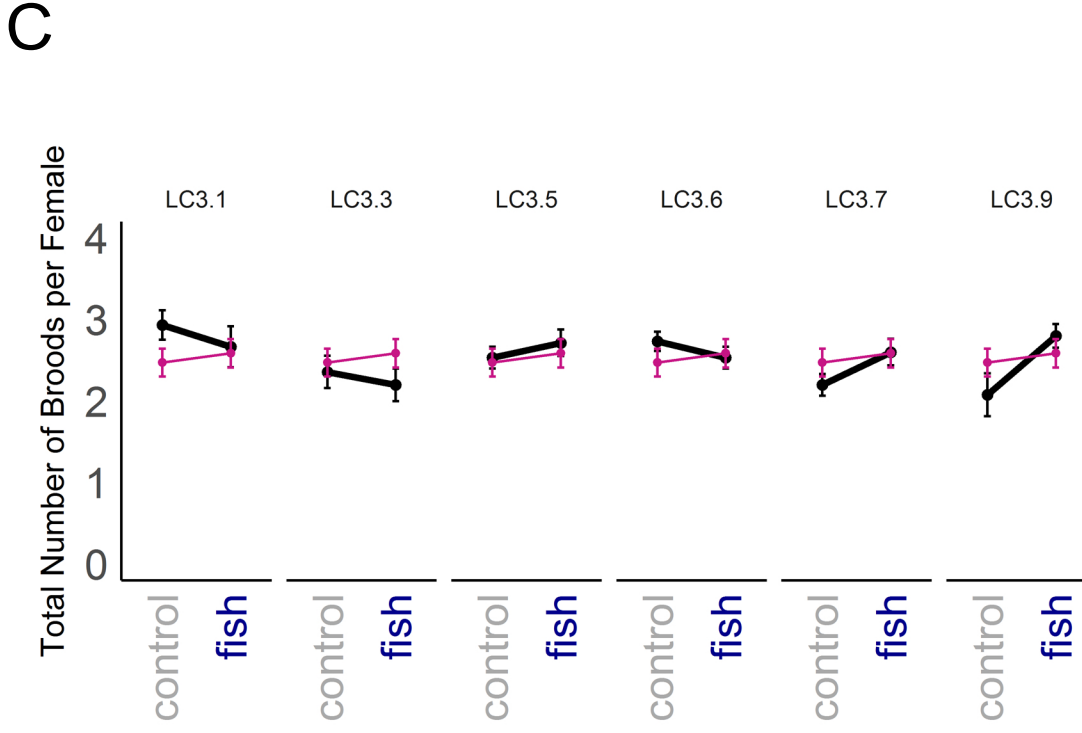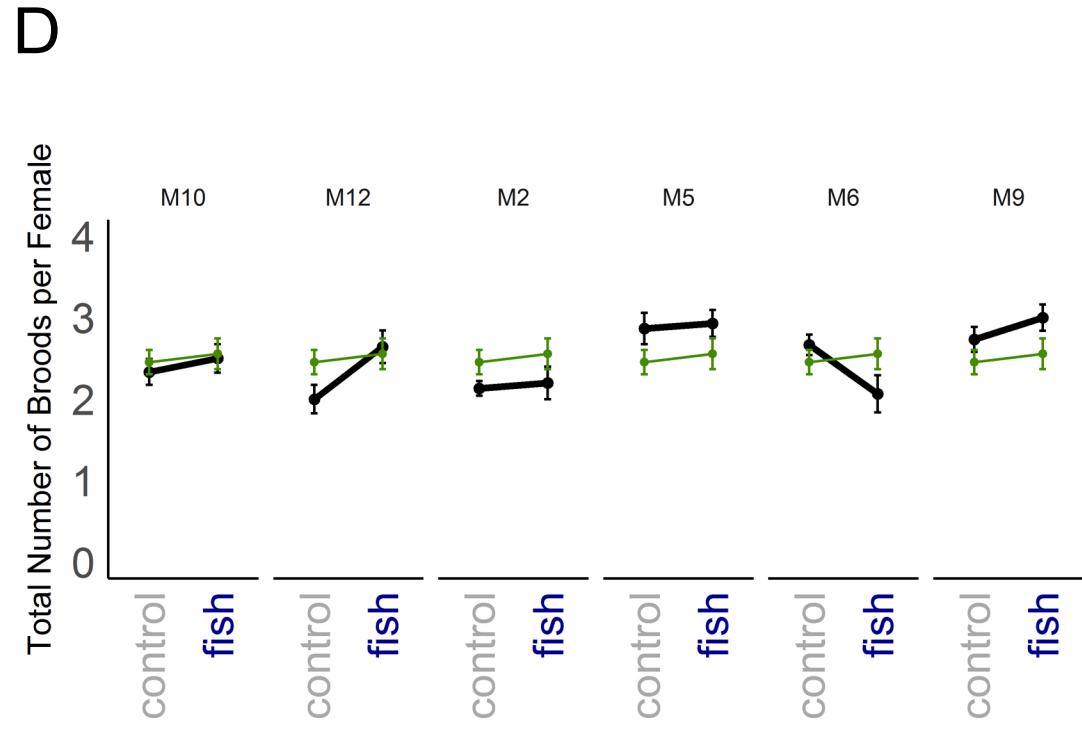

Supplement: Supplemental Information 27 — Genotype mean (+/−SE) within one population are displayed for the trait ‘broods’. The overall within population mean (+/−SE) is displayed in a population specific color. (A) Population Greifensee= popG= ’yellow’. (B) Population Jordan Reservoir= popJ= ’black’. (C) Population Lake Constance= popLC= ’magenta’. (D) Population Müggelsee= popM= ’green’. [file peerj-06-5746-s027.pdf]

A

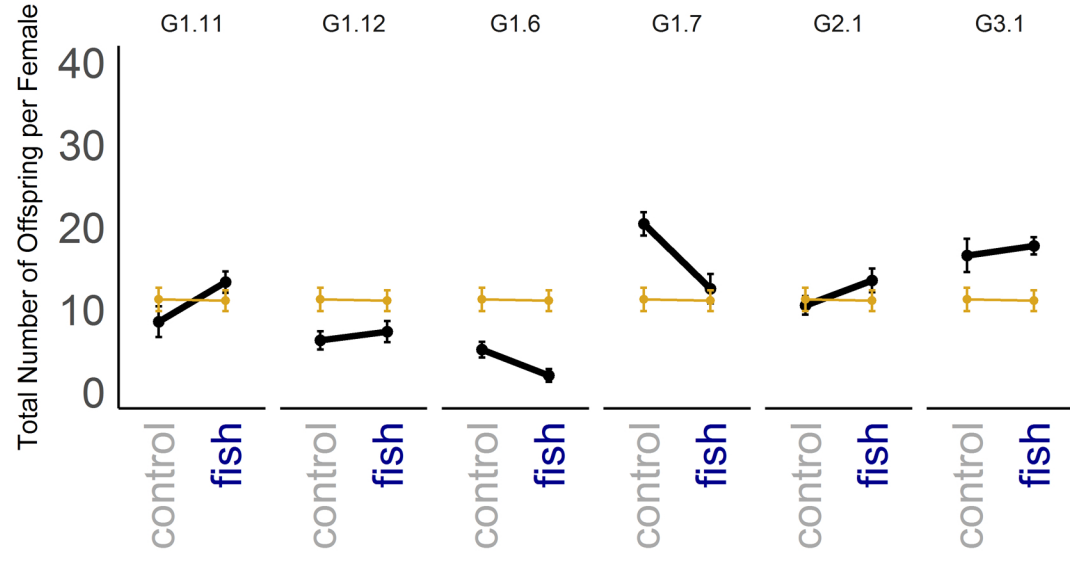

B

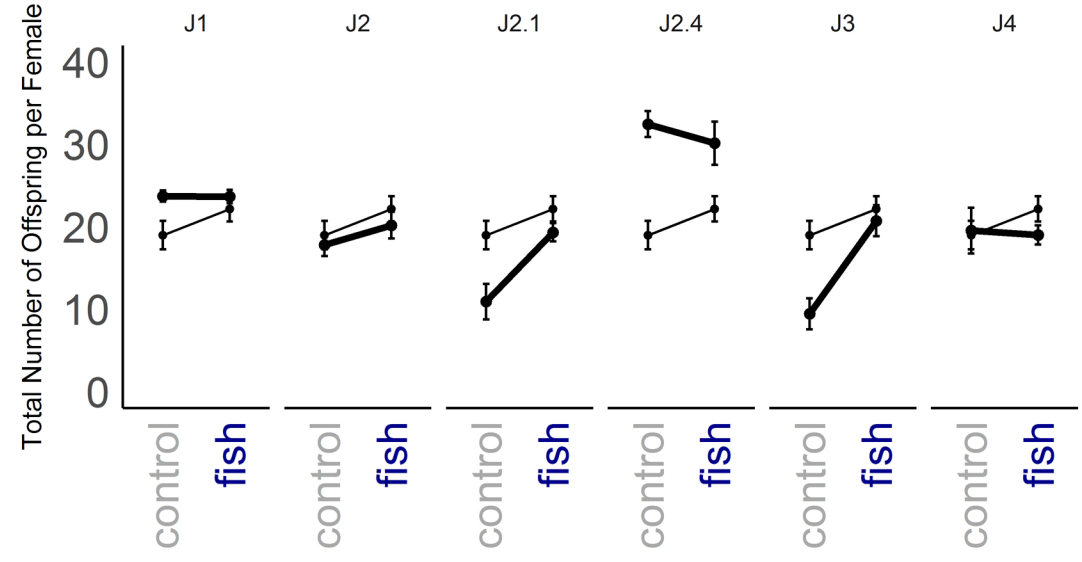

C

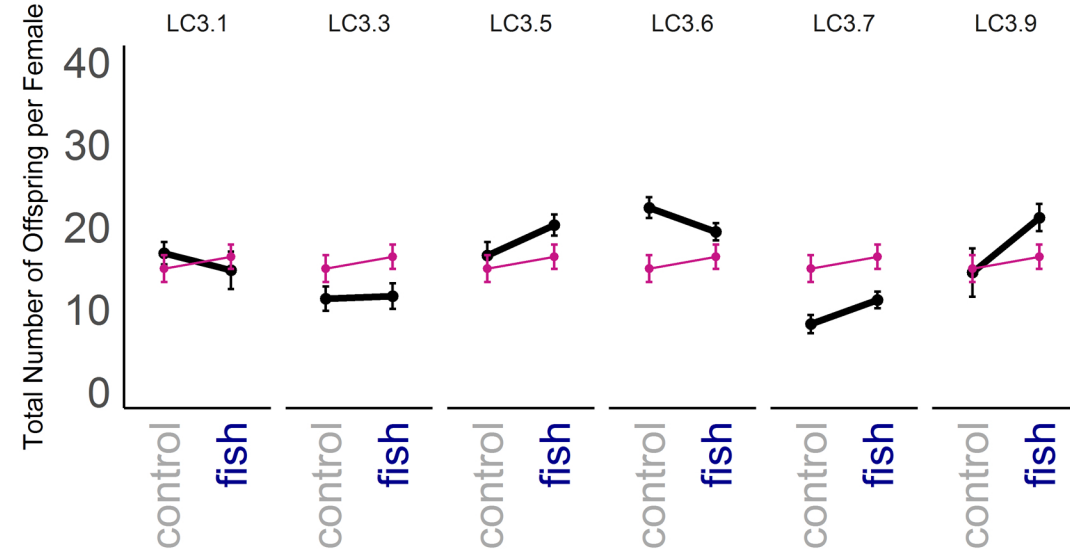

D

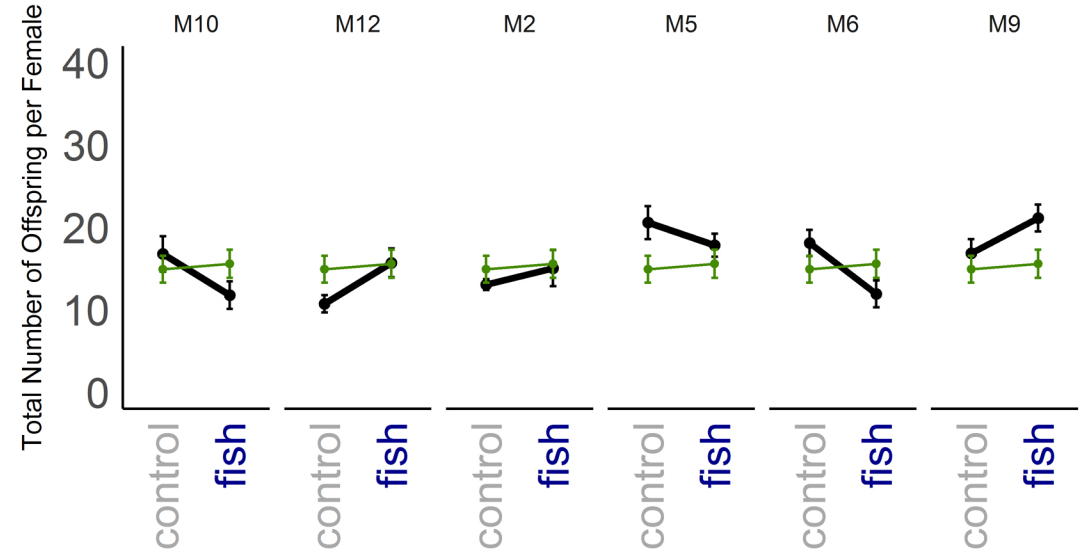

Supplement: Supplemental Information 28 — Genotype mean (+/−SE) within one population are displayed for the trait ‘offspring’. The overall within population mean (+/−SE) is displayed in a population specific color. (A) Population Greifensee= popG= ’yellow’. (B) Population Jordan Reservoir= popJ= ’black’. (C) Population Lake Constance= popLC= ’magenta’. (D) Population Müggelsee= popM= ’green’. [file peerj-06-5746-s028.pdf]

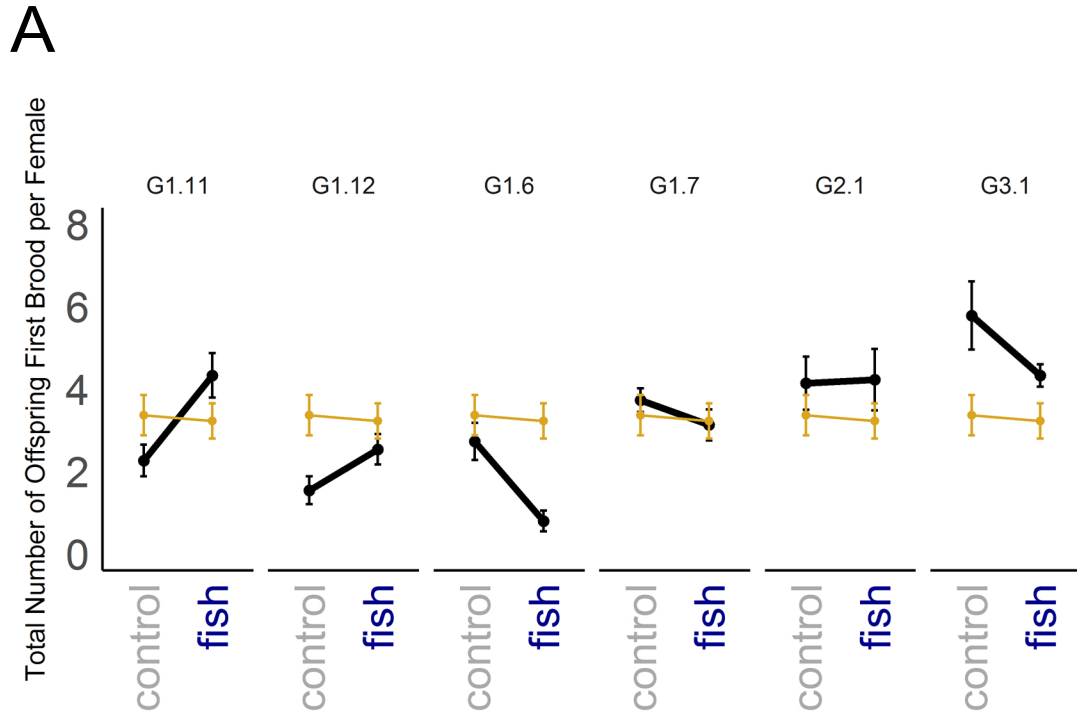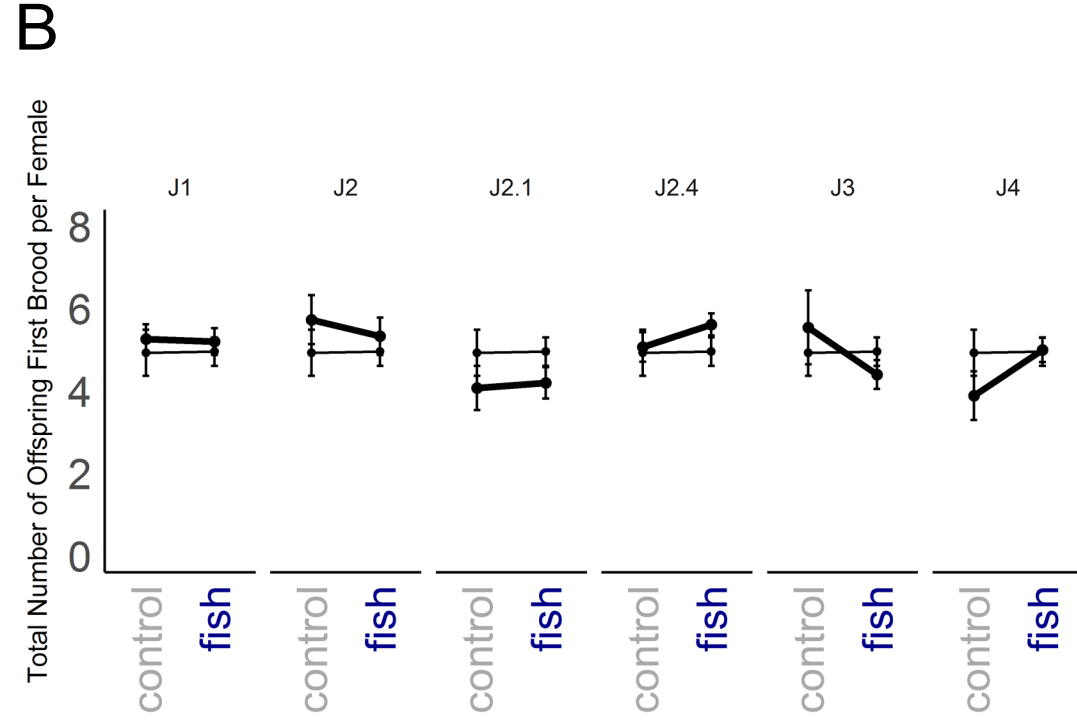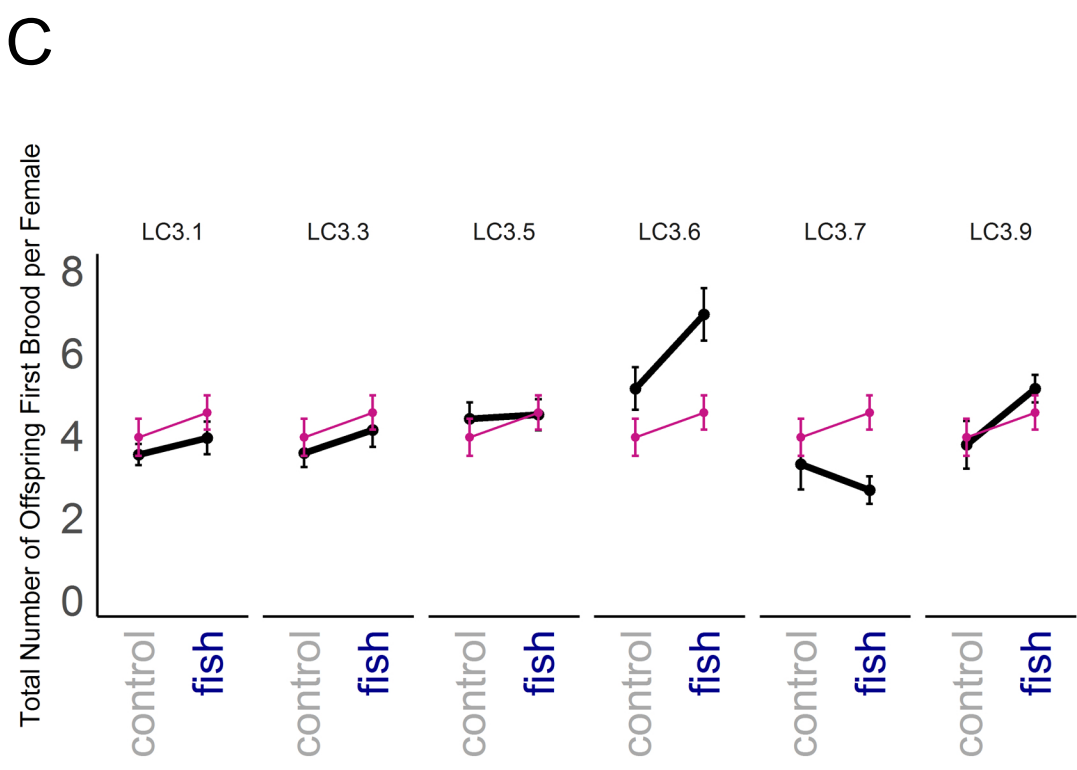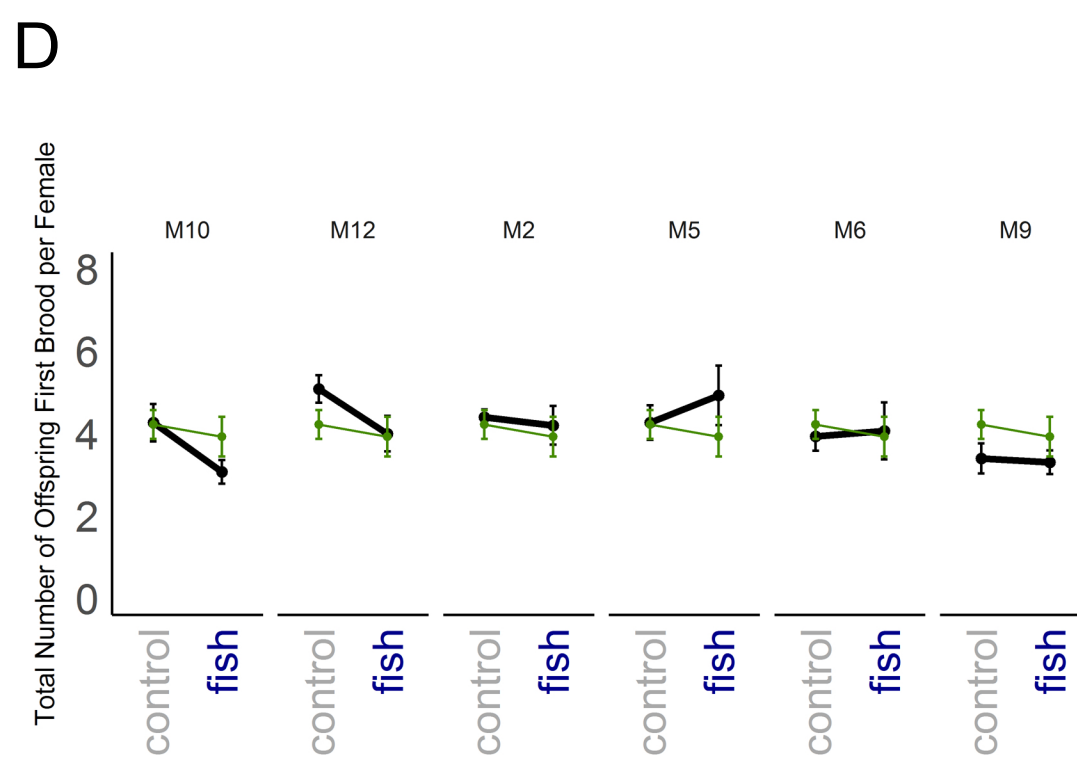

Supplement: Supplemental Information 29 — Genotype mean (+/−SE) within one population are displayed for the trait ‘brood1’. The overall within population mean (+/−SE) is displayed in a population specific color. (A) Population Greifensee= popG= ’yellow’. (B) Population Jordan Reservoir= popJ= ’black’. (C) Population Lake Constance= popLC= ’magenta’. (D) Population Müggelsee= popM= ’green’. [file peerj-06-5746-s029.pdf]

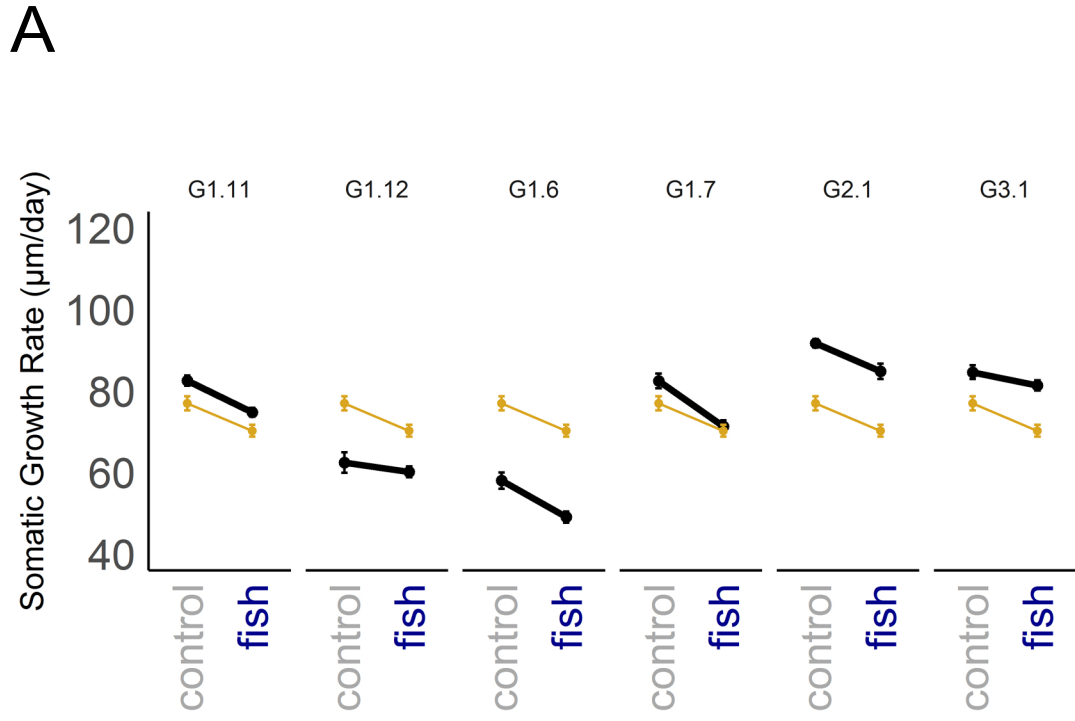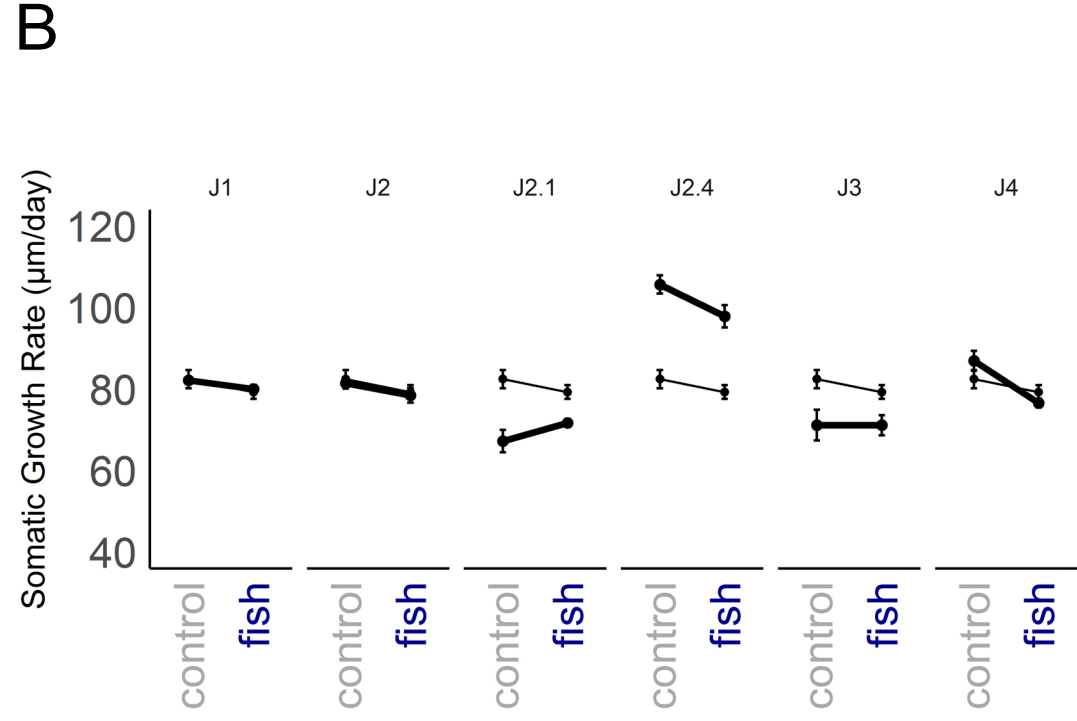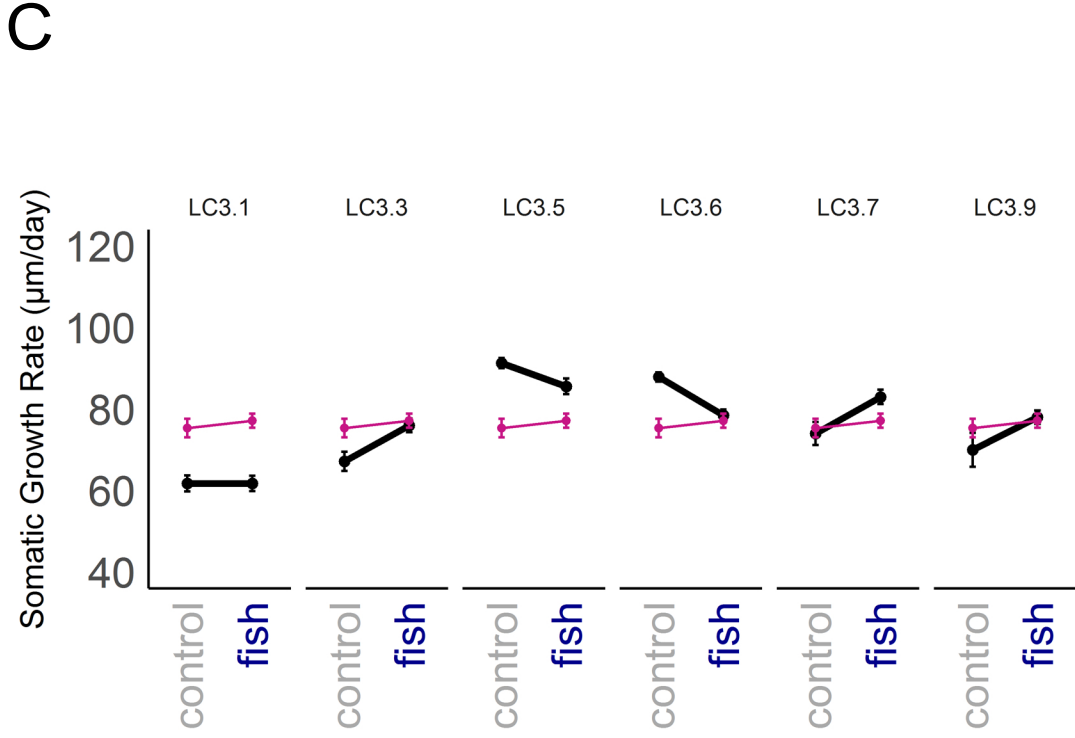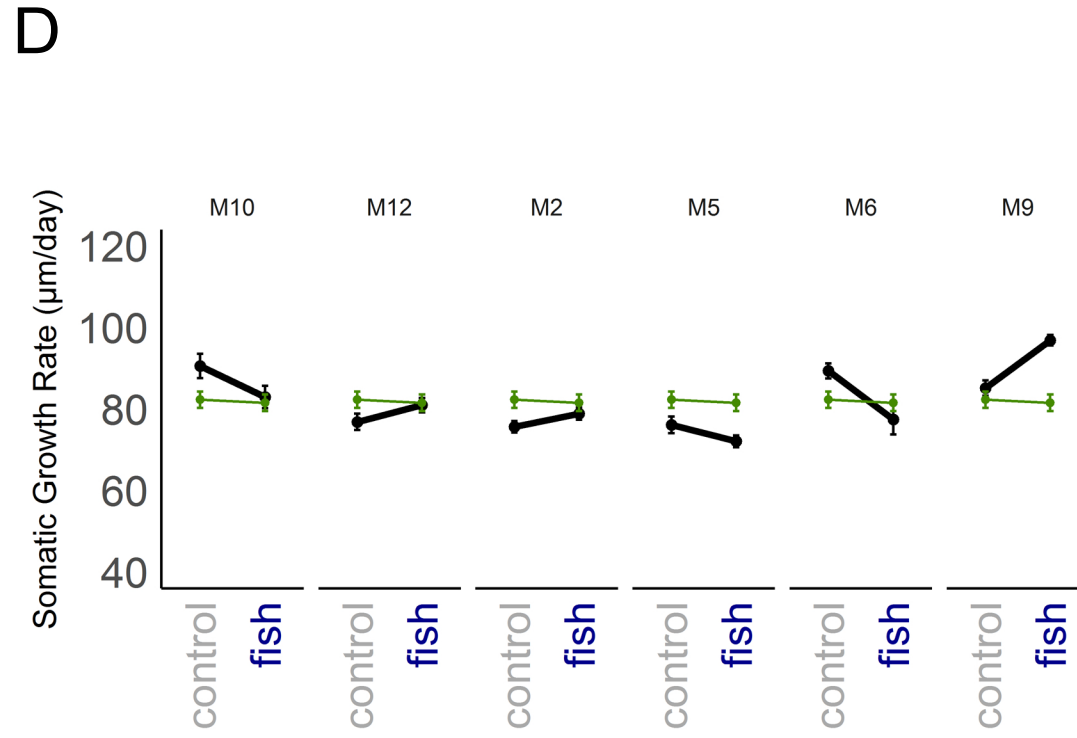

Supplement: Supplemental Information 30 — Genotype mean (+/−SE) within one population are displayed for the trait ‘SGR’ in μm/day. The overall within population mean (+/−SE) is displayed in a population specific color. (A) Population Greifensee= popG= ’yellow’. (B) Population Jordan Reservoir= popJ= ’black’. (C) Population Lake Constance= popLC= ’magenta’. (D) Population Müggelsee= popM= ’green’. [file peerj-06-5746-s030.pdf]

A

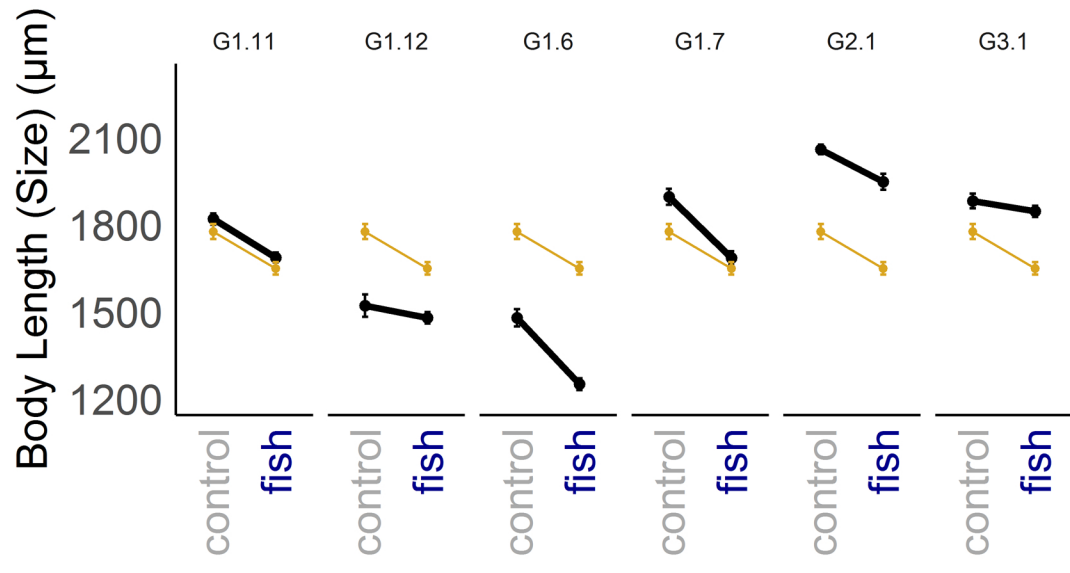

B

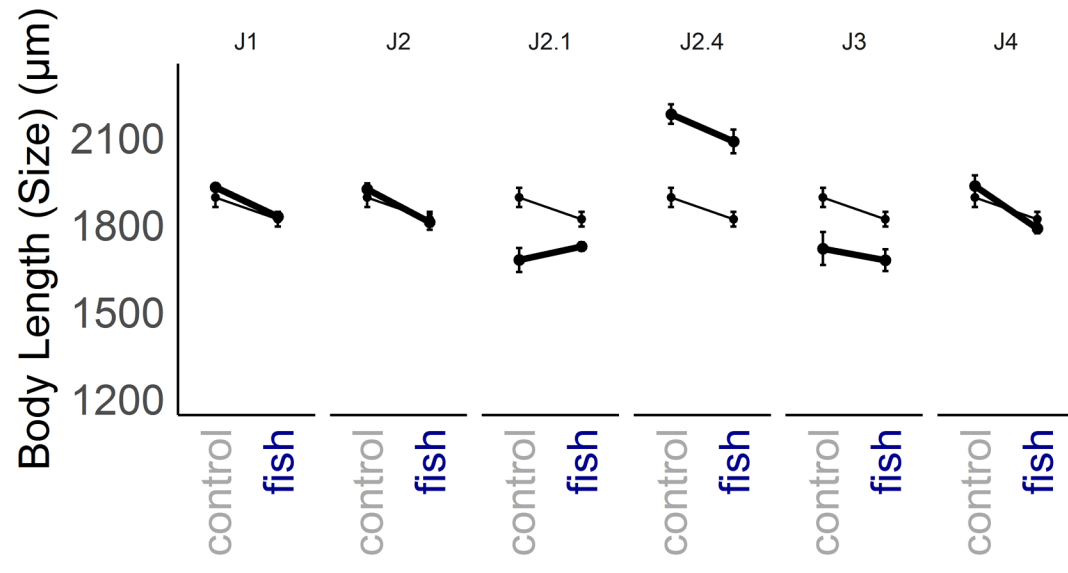

C

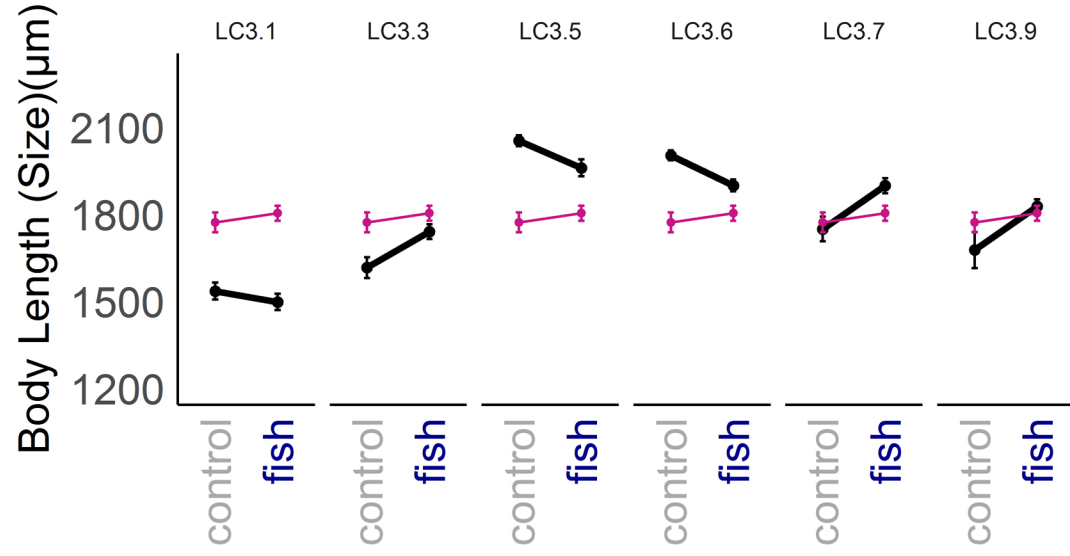

D

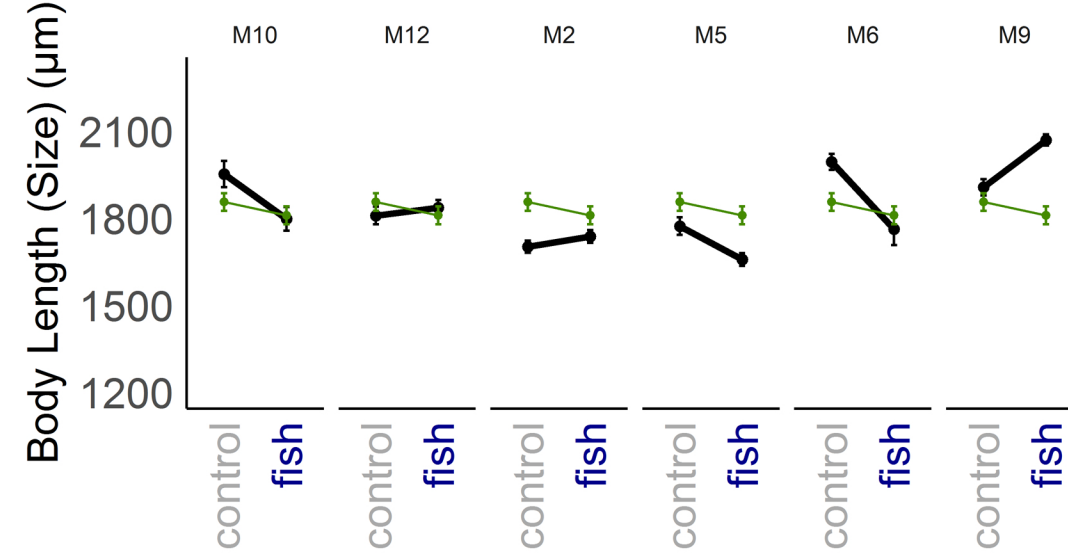

Supplement: Supplemental Information 31 — Genotype mean (+/−SE) within one population are displayed for the trait ‘size’ in μm. The overall within population mean (+/−SE) is displayed in a population specific color. (A) Population Greifensee= popG= ’yellow’. (B) Population Jordan Reservoir= popJ= ’black’. (C) Population Lake Constance= popLC= ’magenta’. (D) Population Müggelsee= popM= ’green’. [file peerj-06-5746-s031.pdf]
